# Multiple challenges of antibiotic use in a large hospital in Ethiopia – a ward-specific study showing high rates of hospital-acquired infections and ineffective prophylaxis

**DOI:** 10.1186/s12913-018-3107-9

**Published:** 2018-05-03

**Authors:** Girma Gutema, Helle Håkonsen, Ephrem Engidawork, Else-Lydia Toverud

**Affiliations:** 1 Department of Social Pharmacy, School of Pharmacy, University of Oslo, P.O. Box 1068, 0316 Oslo, Norway; 2 Department of Public Health and Community Medicine, Institute of Medicine, University of Gothenburg, Gothenburg, Sweden; 3 Department of Pharmacology and Clinical Pharmacy, School of Pharmacy, Addis Ababa University, Addis Ababa, Ethiopia

**Keywords:** Antibiotics, Ceftriaxone, Cephalosporin, Consumption, Ethiopia, Hospital, Tertiary

## Abstract

**Background:**

This project aims to study the use of antibiotics in three clinical wards in the largest tertiary teaching hospital in Ethiopia for a period of 1 year. The specific aims were to assess the prevalence of patients on antibiotics, quantify the antibiotic consumption and identify the main indications of use.

**Method:**

The material was all the medical charts (*n* = 2231) retrieved from three clinical wards (internal medicine, gynecology/obstetrics and surgery) in Tikur Anbessa Specialized Hospital (TASH) in Addis Ababa between September 2013 and September 2014. Data collection was performed manually by four pharmacists.

**Results:**

Each medical chart represented one patient. About 60% of the patients were admitted to internal medicine, 20% to each of the other two wards. The number of bed days (BD) was on average 16.5. Antibiotics for systemic use were prescribed to 73.7% of the patients (on average: 2.1 antibiotics/patient) of whom 86.6% got a third or fourth generation cephalosporin (mainly ceftriaxone). The average consumption of antibiotics was 81.6 DDD/100BD, varying from 91.8 in internal medicine and 71.6 in surgery to 47.6 in gynecology/obstetrics. The five most frequently occurring infections were pneumonia (26.6%), surgical site infections (21.5%), neutropenic fever (6.9%), sepsis (6.4%) and urinary tract infections (4.7%). About one fourth of the prescriptions were for prophylactic purposes. Hospital acquired infections occurred in 23.5% of the patients (353 cases of surgical site infection). The prescribing was based on empirical treatment and sensitivity testing was reported in only 3.8% of the cases.

**Conclusions:**

In the present study from three wards in the largest tertiary teaching hospital in Ethiopia, three out of four patients were prescribed antibiotics, primarily empirically. The mean antibiotic consumption was 81.6 DDD/100BD. Surgical site infections constituted a large burden of the infections treated in the hospital, despite extensive prescribing of prophylaxis. The findings show the need to implement antibiotic stewardship programs in Ethiopian hospitals with focus on rational prescribing, increased sensitivity testing and better procedures to prevent hospital acquired infections.

## Background

The use of antibiotics, as practiced today with the steadily increasing rates of resistance, can be considered the greatest global health problem of our time. Excessive use combined with tourism, migration, and use of antibiotics in agriculture are important driving factors for the spread of antibiotic resistance [1, 2]. In countries with immature healthcare systems antibiotics are among the most commonly used drugs [3–5] and resistance rates can be up to 100% for ordinary pathogenic microbes [6–8]. In urban as well rural areas antibiotics are often bought without a prescription and used both for viral and bacterial infections [9]. It can be difficult to know if the drugs have any effect, especially since most drugs are prescribed empirically without sensitivity testing [10].

In many countries antibiotic stewardship programs have been implemented to promote a more rational use of antibiotics [11]. However, Ethiopia is a country that has no such program in either the public or the private sector [12]. The country has about one hundred million inhabitants, but only one doctor per 35,000 [13]. In the rural areas, the majority of the population never meet any medically trained person [14]. The healthcare system is administratively organised with three levels of hospitals: primary hospitals serving 60,000–100,000 inhabitants, general hospitals serving 1.0–1.5 million inhabitants, and specialized hospitals serving 3.5–5.0 million inhabitants [13]. It is in the general and specialized hospitals that antibiotics are used to a very great extent [3].

In an Ethiopian nationwide survey from 2009 it was found that more than 70% of patients in health centres and hospitals were prescribed between one and six antibiotic agents each [5]. In Gonder University Hospital, the prevalence of antibiotic use was 71% in the surgical ward. Use of broad spectrum agents was found common, often for long periods of time [3]. In Ayder Referral Hospital in Mekelle, it was documented an average point prevalence of 36% antibiotic use in a total of 170 patients in three wards (internal medicine, gynecology/obstetrics and surgery), whereof 81% of the prescribing were found to be inappropriate. Also in this study, the most common reasons were improper duration of treatment and incorrect drug choice [15].

It has been recommended that the aggregated use of antibiotics should be monitored at both local and national levels to better understand the relationship between the use of the drugs and the emerging bacterial resistance [16]. Studies conducted at ward level can generate detailed information about the magnitude and composition of the antibiotic consumption and give an insight into the rationality of prescribing [17]. Furthermore, this would provide information on the real situation of antibiotic utilization at the patient level thereby providing accurate assessments for appropriate regulatory or educational interventions.

Hence, the overall aim of the present project was to study the use of antibiotics in three clinical wards in the largest tertiary teaching hospital in Ethiopia for a period of one whole year. The specific aims were to assess the prevalence of patients on antibiotics, quantify the antibiotic consumption and identify the main indications of use.

## Material

The material for the study consisted of 2231 medical charts retrieved from the wards internal medicine, gynecology/obstetrics and surgery in Tikur Anbessa Specialized Hospital (TASH) – the largest tertiary teaching hospital in Addis Ababa (700 beds) for the period 11th of September 2013 to 10th of September 2014.

## Methods

### Data collection

Data collection was carried out by four hospital pharmacists between January and April 2015. Patient admission and discharge charts were used to manually retrieve all medical charts from the selected wards. A standardized data collection form was developed based on the criteria for drug use evaluation by the World Health Organization (WHO) [18]. It contained the following information: if the patient had been prescribed antibiotics for systemic use (J01class of the WHO Anatomical Therapeutic Chemical (ATC) classification system) during the hospital stay, number of bed days (excluding the day of discharge), number, name(s), strength, dose, administration form (oral or parenteral) and indication of antibiotics prescribed, prescriptions of other drugs, and if sensitivity testing had taken place.

A pilot study was undertaken for the first 45 medical charts (i.e. 15 cases for each ward).

### Data analysis

Epi Info™ 7.1.5 (Center for Surveillance, Epidemiology & Laboratory Services, USA) and SPSS Statistics, version 21 (SPSS Inc., Chicago, IL, USA) were used for data analyses. The number of defined daily doses (DDD) was calculated for each product based on WHO guidelines from 2014 [19]. For products with more than one active antibiotic ingredient, each of these was accounted for separately. The consumption of the various antibiotics was ranked according to the drugs constituting 90% of the overall consumption (referred to as DU90%) [20].

## Results

Each of the 2231 medical charts represented one patient. A majority of patients (57.5%) were admitted to the internal medicine ward, 21.4% to the gynecology/obstetrics ward and 21.1% to the surgery ward. Table 1 shows the distribution of bed days (BD) for patients admitted to the three wards during the study period.

**Table 1:** Distribution of bed days (BD) for patients (*n* = 2231) admitted to three clinical wards at TASH during the one-year study period

Clinical ward	Number of BD (%)	Mean number of BD (sd)	Number of patients with > 30 BD (%)
Internal medicine	25,766 (70.1)	20.1 (17.0)	245 (19.1)
Gynecology/Obstetrics	4974 (13.6)	10.4 (9.9)	54 (11.3)
Surgery	5993 (16.3)	12.7 (16.9)	21 (4.4)
Total	36,733 (100.0)	16.5 (16.3)	320 (14.3)

In all, 1645 patients (73.7%) were prescribed antibiotics for systemic use (hereafter referred to as antibiotics). As can be seen from Tables 1, 320 patients stayed in the hospital for more than 30 days. Of these, 283 patients (88.4%) were prescribed antibiotics. The drugs were primarily prescribed empirically, and sensitivity tests were found to be performed in 3.8% of the cases (Table 2).

**Table 2:** Number of patients in the three clinical wards who were prescribed antibiotics for systemic use (ATC: J01) and number of sensitivity tests performed during the one year study period (*n* = 1645)

Clinical ward	Number of patients prescribed antibiotics (%)	Number of sensitivity tests performed (%)
Internal Medicine	939 (73.2)	54 (5.8)
Gynecology/Obstetrics	310 (64.9)	5 (1.6)
Surgery	396 (84.1)	4 (1.0)
Total	1645 (73.7)	63 (3.8)

There was a great variety of infections being treated. The five most frequently occurring ones were pneumonia (26.6%), surgical site infections (21.5%), neutropenic fever (6.9%), sepsis (6.4%) and urinary tract infections (4.7%). A high proportion of the prescriptions in the gynecology/obstetric ward were for prophylactic use, i.e. 63.6%, compared with 41.3% in surgery and 8.5% in internal medicine. Of the patients who were given prophylaxis in gynecology/obstetric, 44.1% got a surgical site infection. In surgery, 65.3% got a surgical site infection despite being given prophylactic treatment before the operation. Among the 1645 patients who got antibiotics, 63.5% were also prescribed drugs for other conditions.

Five hundred and twenty-seven of the total of 2231 patients (23.5%) in the three wards were prescribed antibiotics because of a hospital acquired infection (HAI). Of the 1645 patients who were prescribed antibiotics, the percentage was 31.9. For the total number of patients who stayed more than 30 days in the hospital (320 patients), the percentage was 46.3. The HAIs were mainly reported as surgical site infection (353 cases), hospital acquired pneumonia (97 cases), cystitis (71 cases) or just ‘HAI’ (6 cases). Of the patients who were diagnosed with a HAI, about two out of three (289) had no other infection.

Table 3 shows the number of antibiotics prescribed to the patients in the different wards. The mean number varied from 1.5 in gynecology/obstetrics to 2.5 in internal medicine. The patients were prescribed between 1 and 9 different agents, with an average of 2.1 per patient. On average for the three wards, 39.1% of the patients were prescribed one antibiotic agent and 33.6% were prescribed two. Overall, 82.4% of the prescribed antibiotics were administered parenterally (intravenously/−muscularly) and 17.6% orally.

**Table 3:** Number of antibiotics prescribed to the patients in the three clinical wards during their hospitalization (*n* = 1645)

	Number of antibiotics per patient	1	2	3	4	5–9	Mean (sd)
Number of patients (%)	Internal Medicine	194 (20.7)	383 (40.8)	189 (20.1)	94 (10.0)	79 (8.4)	2.5 (1.3)
	Gynecology/Obstetrics	213 (68.7)	53 (17.1)	26 (8.4)	11 (3.5)	7 (2.3)	1.5 (1.0)
	Surgery	236 (59.6)	117 (29.5)	27 (6.8)	9 (2.3)	7 (1.8)	1.6 (0.9)

The one-year consumption of antibiotics was 91.8 DDD/100 BD in internal medicine, 71.6 DDD/100 BD in surgery and 47.6 DDD/100 BD in gynecology/obstetrics. The average number for the three wards together was 81.6 DDD/100 BD. The number of DDDs per patient was 24.8, 10.9 and 7.6 for the three wards, respectively (Table 4).

**Table 4:** Consumption in DDD/100BD and DDD/patient by class of antibiotics for systemic use (ATC: J01) in the three clinical wards at TASH during the one-year study period (*n* = 1645)

Class of antibiotics	ATC Code	Antibiotic consumption					
		Internal Medicine		Gynecology/Obstetrics		Surgery	
		DDD/100BD	DDD/patient	DDD/100BD	DDD/Patient	DDD/100BD	DDD/patient
Third-generation cephalosporins	J01DD	33.1	9.1	10.6	1.7	38.5	5.8
Fluoroquinolones	J01MA	11.4	3.1	1.8	0.3	1.9	0.3
Imidazole derivatives	J01XD	11.3	3.1	7.2	1.1	16.3	2.5
Glycopeptides	J01XA	8.9	2.4	1.1	0.2	5.2	0.8
Sulfonamides and trimethoprim	J01EE	7.8	2.1	0.9	0.1	0.2	–
Macrolides	J01FA	5.5	1.5	3.4	0.5	0.3	0.1
Penicillins with extended spectrum	J01CA	4.1	1.1	16.3	2.6	1.5	0.2
Fourth-generation cephalosporins	J01DE	3.1	0.9	–	–	1.3	0.2
Penicillins plus beta-lactamase inhibitors	J01CR	2.0	0.5	0.5	0.1	1.0	0.2
Beta-lactamase-sensitive penicillins	J01 CE	1.6	0.4	0.7	0.1	–	
Other aminoglycosides	J01GB	0.9	0.2	1.3	0.2	0.8	0.1
Lincosamides	J01FF	0.6	0.1	–	–	0.3	0.1
Beta-lactamase-resistant penicillins	J01CF	0.6	0.2	0.4	0.1	4.0	0.6
Tetracyclines	J01AA	0.3	0.1	2.3	0.4	–	–
Carbapenems	J01DH	0.2	–	–	–	–	–
First-generation cephalosporins	J01DB	0.2	0.1	1.1	0.2	0.2	–
Streptomycins	J01GA	0.1	–	–	–	–	
Amphenicols	J01BA	0.1	–	–	–	0.1	–
Total	J01	91.8	24.8	47.6	7.6	71.6	10.9

**Fig. 1:**
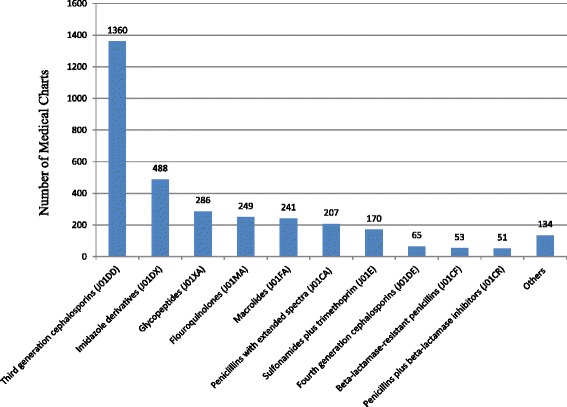
The number of patients who were prescribed different antibiotics for systemic use (ATC: J01) during the one-year study period

Figure 1 shows the number of patients who were prescribed different classes of antibiotics one or more times. As can be seen, third-generation cephalosporins (J01DD) were prescribed to 82.7% of the patients.

**Fig. 2:**
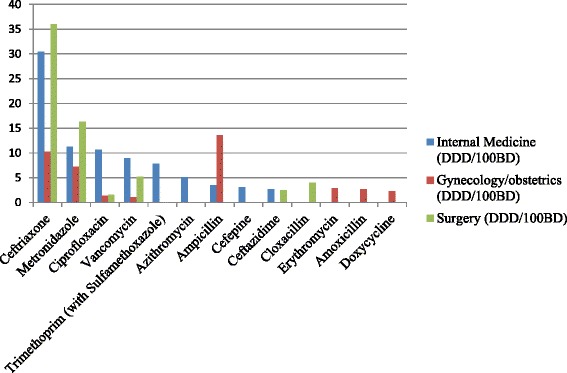
Antibiotics constituting the drug utilization 90% (DU90%) in three clinical wards at TASH (*n* = 1645)

Nine antibiotic agents constituted 90% of the antibiotic consumption (DU90%) in the internal medicine and gynecology/obstetrics wards. In surgery, six products accounted for the DU90% (Fig. 2). Four antibiotic products were present among the DU90% in all three wards: ceftriaxone (76.7 DDD/100BD), metronidazole (34.8 DDD/100BD), vancomycin (15.2 DDD/100BD) and ciprofloxacin (13.7 DDD/100BD). Ceftriaxone was the most commonly prescribed antibiotic in surgery (36.0 DDD/100BD) and internal medicine (30.4 DDD/100BD), and ampicillin was the most prescribed antibiotic in gynecology/obstetrics (13.6 DDD/100BD).

## Discussion

In the present study from three clinical wards (internal medicine, gynecology/obstetrics and surgery) in the largest tertiary teaching hospital in Addis Ababa, Ethiopia, it was found that three out of four patients were prescribed antibiotics, of whom almost 90% were prescribed a broad spectrum agent (a 3rd or 4th generation cephalosporin). One in four patients was prescribed antibiotics for treatment of an infection acquired in the hospital – a proportion which is higher than what have been found in previous studies from Ethiopian hospitals [8, 21]. Particularly, the considerable number of surgical site infections, despite the high use of prophylaxis, shows how challenging treatment has become primarily in terms of antibiotic resistance.

The present study found an average antibiotic consumption of 81.6 DDD/100BD in the three clinical wards, varying from 47.6 DDD/100BD in gynecology/obstetrics to 91.8 DDD/100BD in internal medicine. In a study from different hospitals in Lebanon [22] the overall antibiotic consumption was documented to be 72.6 DDD/100BD, which can be seen as in the same order of magnitude as the present results. As an example from Europe, where stewardship programs often exist and resistance rates are lower, Norwegian hospitals had in 2016 a mean consumption of 76 DDD/100BD which was a 24% increase from 2006 [23]. In European context, Norway is among the top 10 countries with the lowest consumption of antibiotics. Furthermore, a meta-analysis of 80 studies from different hospital settings worldwide reported an average antibiotic consumption of 58.6 DDD/100BD [24]. A sub-analysis of the average consumption in medical wards found 67.7 DDD/100BD. Most of the included studies were performed in tertiary, teaching or public hospitals, predominantly in Western Europe and in Mediterranean countries. No studies from Africa were identified [24].

On average, 2.1 antibiotics were prescribed per patient with a range from 1.5 to 2.5 in the three wards. The number was highest in internal medicine where the patients had the longest stays and therefore a great risk of HAI. In surgery, where the average number was 1.6, we found the highest prevalence of patients on antibiotics. This must be seen in relation to the extensive use of prophylaxis and the extent of resistance which probably make it necessary to treat a high number of SSIs. Also in the gynecology/obstetrics ward, prophylactic use of antibiotics contributed significantly to the consumption. This can to some extent be explained by a high number of caesarean deliveries [25], where ampicillin is recommended as prophylaxis [26]. Moreover, ampicillin is part of the standard regimen recommended for the treatment of several gynecological infections [27, 28].

In the internal medicine and surgery wards, third-generation cephalosporins were clearly the most commonly prescribed class of antibiotics. Ceftriaxone alone constituted 50% of the overall antibiotic consumption in the surgery ward whereas the proportion of that drug was 33% in internal medicine. In comparison, the report from the Norwegian hospitals showed that the cephalosporins accounted for 17% of the total consumption [23]. The extensive use of ceftriaxone has previously been the focus of studies from Ethiopian hospitals including TASH [10, 29]. In a study from the internal medicine and emergency wards at TASH, it was found that almost half of the patients received ceftriaxone when it was not indicated as first-line treatment [10]. In spite of its broad use, it is now well-known that ceftriaxone is another antibiotic agent with scarce effect due to high resistance rates [8].

### Methodological considerations

A major strength of the present study is the comprehensive data from medical charts which was manually collected for a whole year. The use of medical charts in studies of this kind is recommended because they provide prescription data at the individual patient level. However, a likely limitation is the extent of incomplete reporting of data in the medical charts. For example, it was not noted if a patient had been transferred from one ward to another, but available information suggests this is something that rarely happens. In addition, since the chart follows the patient during transfer, the move would not be counted as a multiple admission. We applied the DDD per 100BD as the unit of measurement of antibiotic consumption, which can be used for national and international comparisons [19]. Nevertheless, it is generally challenging to compare studies between countries, because of lack of comparable data, different study methodologies and, in this case, hospital settings [24, 30]. Since Ethiopia, like other African countries, struggles with the alarmingly high rates of antibiotic resistance it can easily lead to prescribing of higher doses, prolonged treatment, or change of drug. The high prevalence of empirical treatment does not ameliorate the situation.

## Conclusion

In the present study from three wards in the largest tertiary teaching hospital in Ethiopia, three out of four patients were prescribed antibiotics, primarily empirically. The mean antibiotic consumption was 81.6 DDD/100BD. Surgical site infections constituted a large burden of the infections treated in the hospital, despite extensive prescribing of prophylaxis. The findings show the need to implement antibiotic stewardship programs in Ethiopian hospitals with focus on rational prescribing, increased sensitivity testing and better procedures to prevent hospital acquired infections.

## Abbreviations

ATC: Anatomical Therapeutic Chemical (classification system)
BD: Bed days
DDD: Defined daily doses
DU90%: Drug utilization 90%
HAI: Hospital acquired infection
SSI: Surgical site infection
TASH: Tikur Anbessa Specialized Hospital
WHO: World Health Organization
